# Modulating Protein Glycation in Skim Milk Powder via Low Humidity Dry Heating to Improve Its Heat-Stabilizing Properties

**DOI:** 10.3390/foods14244197

**Published:** 2025-12-06

**Authors:** Zijun Zhao, Riza Flores, Bruno De Meulenaer, Paul Van der Meeren

**Affiliations:** 1 Particle & Interfacial Technology Research Group, Department Green Chemistry & Technology, Faculty of Bioscience Engineering, Ghent University, B-9000 Ghent, Belgium; zijun.zhao@ugent.be (Z.Z.);; 2 Nutrition and Food Chemistry Research Group, Department Food Technology, Safety and Health, Faculty of Bioscience Engineering, Ghent University, B-9000 Ghent, Belgium; bruno.demeulenaer@ugent.be

**Keywords:** skim milk powder, Maillard reaction, low humidity, high temperature, recombined filled evaporated milk emulsion, heat stability

## Abstract

The limited heat stability of skim milk powder (SMP) constrains its application in high-temperature processes. While dry heating can improve its thermal resistance, it often accelerates the advanced Maillard reaction, compromising protein quality. This study applied low relative humidity conditions (<10% RH) during dry heating to modulate the Maillard reaction, aiming to enhance the heat resistance of SMP and derive recombined filled evaporated milk emulsions with fewer undesirable changes in colour and solubility. SMP was subjected to dry heating at 80, 100, and 120 °C for durations ranging from 2 to 20 min (at 120 °C) and up to 16 h (at 80 °C). The progression of the Maillard reaction and associated protein modifications were evaluated. The results indicate that the advanced Maillard reaction was retarded, evidenced by minimal colour development and well-preserved protein solubility (90–97%, *n* = 3), determined using the Lowry assay on the supernatants. The hydroxymethylfurfural and protein carbonyl contents increased only moderately with temperature and time. Moreover, the sulfhydryl group content remained largely stable, consistent with limited disulfide-mediated aggregation. Heat treatment of SMP at 120 °C for 10 min greatly improved its heat stability, as reflected by a 25-fold reduction in the volume-weighted average diameter (*D_4,3_*; 95% CI = 3 to 47) and a 108-fold reduction in the consistency coefficient (*K*; 95% CI = 12 to 200) of the SMP-derived sterilised recombined filled evaporated milk (RFEM) compared to the control. These findings demonstrate that dry heating under low RH helps to improve the functional properties of SMP without inducing the detrimental effects associated with advanced Maillard products.

## 1. Introduction

Skim milk powder (SMP) is generally used for producing recombined filled evaporated milk emulsions (RFEM), a concentrated dairy-derived product [1]. Pasteurisation and sterilisation processes are often applied to milk products to enhance their shelf-life and safety. Compared to non-concentrated dairy drinks, RFEM is more susceptible to heat-induced coagulation as it contains a higher amount of heat-labile whey proteins. The coagulation in RFEM after sterilisation is mainly due to the interaction between denatured whey proteins and casein micelles, which severely impairs the product quality and production equipment [2]. During sterilisation, whey proteins first unfold and release a free sulfhydryl group (-SH), which can then interact with κ-casein, leading to flocculation and/or aggregation.

Heat-induced glycation between proteins and lactose in SMP has been proven to be a feasible way to improve their emulsifying and heat-stabilising properties via protein modification, providing extra steric hindrance against heat-induced protein aggregation [3,4]. Glycation occurs in the early stage of the Maillard reaction (MR). However, upon processing and storage, early-stage Maillard reaction products (glycated proteins) may undergo subsequent reactions where intermediate or advanced Maillard reaction products are formed, and protein crosslinking is induced, causing poor solubility and brown colour [5].

It is important to monitor and control the MR to obtain ideal heat-stable glycated products. Temperature and humidity are key factors in modulating the MR [6]. Higher temperatures promote the accumulation of Maillard reaction products. Liu and Zhong [7] demonstrated that elevated dry heat temperatures significantly shorten the time required to enhance the heat stability of glycated whey protein solutions while also limiting colour development. On the contrary, water activity exhibits a dual effect on modulating the MR. The MR is favoured at a_w_ values of 0.6–0.7 in milk powder [8]. A high a_w_ value (>0.7) could lead to a dilution effect, slowing down the MR, while the reduced mobility of reactants in a dry system also retards the MR. Phosanam et al. (2020) found that low-humidity conditions (11% RH) greatly reduced colour change and caking strength compared to conditions of 44% and 85% RH after 21 days of storage at 25, 35, and 45 °C [9]. Although these storage temperatures are lower than those used in typical dry-heating processes, their findings support the hypothesis that low humidity may retard unwanted reactions, including the advanced Maillard reaction, protein oxidation, and protein cross-linking during dry heating to some extent. Additionally, compared to incubating SMP at humid conditions, which may make it highly susceptible to mould growth, a low-humidity atmosphere is more practical, especially for long-term storage.

While dry heating at 60 °C and 74% RH has been frequently performed in previous studies, the application of high temperatures combined with low humidity to SMP remains largely unexplored. The effects of temperature on SMP properties and the MR under such low-humidity conditions are unknown. Low RH may retard the MR at lower temperatures but could be ineffective beyond a specific threshold, potentially the glass transition temperature (Tg) at that RH. Furthermore, different stages of the MR in SMP may respond differently to these combinations. An operational window likely exists for each temperature to balance early glycation against advanced MR, which may become narrower at higher temperatures.

In this study, dry heating at a low relative humidity (<10% RH) in combination with high temperatures (80, 100, and 120 °C) was applied to SMP. We supposed that this completely dry condition could reduce unwanted changes from the MR, whereas a high temperature should promote glycation between proteins and lactose in a short time and improve the heat stability of SMP-derived RFEM. To assess the extent of the Maillard reaction, the intermediate MR product, hydroxymethylfurfural (HMF), was detected using high-performance liquid chromatography (HPLC), and browning was evaluated by CIE Lab colorimetry. Moreover, the sulfhydryl and protein carbonyl contents were determined to quantify disulfide bond formation and protein carbonyls, respectively. Finally, the physicochemical properties of the dry-heated samples were evaluated, including the solubility of SMP and the heat stability of the derived RFEM, by comparing its droplet size and consistency before and after sterilisation at 120 °C for 30 min.

## 2. Materials and Methods

### 2.1. Materials

Low-heat SMP, characterised by a whey protein nitrogen index (WPNI) of 6.44, was obtained from Milcobel Dairy Corporation (Kallo, Beveren, Belgium). According to the certificate of analysis of the manufacturer, the SMP contained 33.1% of proteins and 0.64% of fat (*w*/*w*). Fresh refined sunflower oil (Boni, Halle, Belgium) was obtained from a local supermarket.

### 2.2. Dry Heat Treatment of SMP

Silica gel (1 kg) was first incubated in an oven at 120 °C overnight, which was assumed to be completely dry and to provide a low relative humidity (RH < 10%), as verified by a digital thermometer (ThermoPro^®^ TP50 Digital LCD Thermometer, Dubai, United Arab Emirates). A desiccator (10 L) containing this completely dry silica gel was placed into an oven (Universal Oven Memmert UN-55, Schwabach, Germany) and left overnight for equilibration. The temperature of the oven was set at 80, 100, and 120 °C, respectively. Ten grams of SMP was spread in an aluminium Petri dish (diameter = 8.80 cm; thickness of the SMP layer = 0.30 cm) and then transferred into the desiccator. The distance from the desiccant to sample was 5.00 cm. The incubation time of the SMP at the different temperatures was evaluated in initial trials, and the results are presented in Table 1. It should be noted that the set dry-heating conditions referred to the environment of the desiccators before transferring SMP, and it takes time for SMP to reach the set temperature and humidity. Following incubation and cooling to ambient temperature, the dry-heated SMP was ground with a pestle and mortar to yield a uniform powder. Based on our experimental findings, although post-heating crushing broke caked SMP into smaller particles, it did not alter the protein solubility of the powder. Incubation of SMP was performed in three independent replicates conducted at different times. SMP without dry heat incubation was set as control.

### 2.3. Colour Development

The colour of samples was measured from the top of the Petri dish using a colorimeter (Minolta Model CM-2500D Spectrophotometer, Konica Minolta Sensing Inc., Osaka, Japan) equipped with a D65 illuminant and 10° standard observer. The spectrophotometer was calibrated with the supplied white calibration tile. For each sample, ten readings at three different positions were collected, with the Petri dish rotated between readings to account for surface heterogeneity. The mean L*, a*, and b* values were used for analysis. The total colour difference (Δ*E*) was calculated using Equation (1),

(1) $$∆E=\sqrt{{\left(L^{*}-L^{0}\right)}^{2}+{\left(a^{*}{-a}^{0}\right)}^{2}+{\left(b^{*}-b^{0}\right)}^{2}}$$

where L*, a*, and b* were the colour parameters of the incubated SMP, whereas *L*^0^, *a*^0^, and *b*^0^ were the colour parameters of the untreated SMP.

### 2.4. Determination of Total HMF

An amount of 5 ml of SMP dispersion (10 wt%) was added into a 50 mL volumetric flask. After adding 5 mL of 0.3 M oxalic acid, the solution was incubated for 60 min in a boiling water bath without a glass stopper; the flask was swirled a few times during incubation. Later, these flasks were cooled down with tap water, and 5 mL of 40% TCA was added to precipitate the protein. After 5 min, distilled water was added until the 50 mL mark. The diluted solution was mixed upside down and then filtered through a 0.45 µm HPLC filter before detection at a 285 nm wavelength using an UPLC UltiMate 3000 system (ThermoFischer Scientific, Dilbeek, Belgium) equipped with a Lichrosorb RP18-5 column (250 mm × 4.6 mm; 5 µm). The isocratic mobile phase contained water/MeOH (90/10, *v*/*v*). The flow rate was set as 1.0 mL/min for 15 min. The injection volume was 20 μL. An external calibration curve in the range of 0.3–25.0 μg/mL was used for quantification, with coefficients of determination (R^2^) ≥ 0.99. The recovery was 90–110%, whereas the LOD and LOQ were 5 ng/mL and 15 ng/mL, respectively. A matrix-matched calibration was used. Standards were prepared in the same manner as the samples.

### 2.5. Protein Solubility of SMP

SMP samples, with or without dry heat treatment, were dispersed in distilled water to a final concentration of 5% (*w*/*v*). The aqueous dispersions were stirred for at least 1.5 h and stored at 4 °C overnight to ensure complete hydration. Following hydration, the dispersions were centrifuged at 700× *g* for 10 min (Sigma 1-15P, Osterode, Germany) to precipitate insoluble protein, as previously described [10]. The protein content of both the aqueous dispersion (before centrifugation) and the supernatant was determined using a modified Lowry assay [11]. Bovine serum albumin (BSA) was used as the protein standard. Protein solubility was subsequently calculated using Equation (2).

(2) $$Protein\;solubility\;(\%)=\frac{C_{s}}{C_{o}}\times 100$$

In Equation (2), *C_s_* is the concentration of milk protein in the supernatant of SMP dispersions after centrifugation, whereas *C_o_* is the concentration of milk protein in the original SMP dispersions before centrifugation.

### 2.6. Determination of Free Sulfhydryl Group (-SH) in SMP

Free sulfhydryl group content in SMP was determined according to Clare et al. (2005) with modifications [12]. Briefly, 0.5 mL of a 17.6% (*w*/*w*) SMP dispersion was diluted in 2.5 mL of 50 mM Tris-HCl buffer (pH 8.0) containing 8 M urea and 50 mM EDTA. An amount of 10 mM of 5,5-dithiobis nitro-benzoic acid (DTNB) was prepared using the Tris-HCl buffer mentioned above. The absorbance of the diluted samples was measured at 412 nm before and after adding 20 μL DTNB against a blank (Tris-HCl buffer). The cuvette path length was 1 cm. The free sulfhydryl content (µmol/g) was calculated according to the following equation [12]:

(3) $$-SH\;content=\frac{A_{after}-A_{before}}{13600\;M^{-1}{\mathrm{cm}}^{-1}}\times 10^{6}\times V÷m_{protein}$$

*A_before_* and *A_after_* represent the absorbance 412 nm before and after DTNB addition and incubation, respectively; *V* is the total volume, i.e., 3.02 mL; and *m_protein_* is the mass of milk protein in 0.5 mL of SMP dispersions, i.e., 0.0293 g.

### 2.7. Protein Carbonyl Content in SMP

The protein carbonyl content in SMP was determined using a method according to Kerkaert et al. [13]. Briefly, 0.4 mL of 2,4-dinitrophenylhydrazine (DNPH) (10 mM in 2 M HCl) was mixed with 0.3 mL of diluted SMP dispersion (1.76%, *w*/*w*). Following a 60 min incubation period in the dark, 0.7 mL of TCA (20%, *w*/*v*) was added. The mixture was incubated on ice for 10 min and then centrifuged at 10,000 rpm for 3 min. The resulting protein pellets were washed three times with 1 mL of ethanol/ethyl acetate (1:1, *v*/*v*) to remove excess DNPH. Finally, the pellets were dissolved in 0.7 mL of 6 M urea (in 20 mM phosphate buffer, pH 2.3), and the absorbance was measured at 370 nm after blank subtraction. Carbonyl content (nmol/mg protein) was calculated as follows [14]:

(4) $$\mathrm{Carbonyl}\;\mathrm{content}\;(\mathrm{nmol}/\mathrm{mg}\;\mathrm{protein})\;=\;\frac{A\times 10^{9}}{22000}\times V÷m_{protein}$$

*A* refers to absorbance at 370 nm; *V* is the total volume, i.e., 0.7 × 10^−3^ L; and *m_protein_* is the mass of milk protein in 0.3 mL of SMP dispersions, i.e., 1.74 mg.

### 2.8. Heat Stability of RFEM Emulsions

#### 2.8.1. Preparation of RFEM Emulsions

SMP was dispersed in 0.02% NaN_3_ aqueous solution, and sunflower oil was added to prepare emulsions containing 16.5% (*w*/*w*) SMP and 6.5% (*w*/*w*) oil. Pre-homogenisation was conducted with a high-speed blender (IKA Ultra-Turrax TV45, Janke & Kunkel, Staufen, Germany) for 1 min, followed by high-pressure homogenisation using a Microfluidizer 110S (Microfluidics Corporation, Newton, MA, USA) at 55 °C. Temperature control was provided by a Julabo heating circulator with open bath CD-B19 (Julabo Labortechnik, Seelbach, Germany). The microfluidizer was operated at a compressed air pressure of 0.2 MPa, corresponding to a liquid pressure of about 28 MPa according to the manufacturer’s pressure conversion chart. The operation lasted for 2 min, corresponding to about 12 passes through the Y-type interaction chamber. After cooling to room temperature, all emulsions were adjusted to a uniform pH of 6.4 to eliminate heat-induced pH shifts. They were then stored at 4 °C overnight to achieve ionic equilibrium prior to the following heat stability test.

#### 2.8.2. Heat Stability Test

An amount of 10 mL of the prepared emulsion was transferred into a 20 mL headspace vial (75.5 mm × 22.5 mm, 1st hydrolytic class; Grace, Deerfield, IL, USA) with an aluminium crimp seal and immersed in an oil bath (120 ± 2 °C) (Fritel turbo SF, 5 L capacity, Vanden Borre, Gent, Belgium). The oil level in the bath was sufficient to fully immerse the RFEM emulsions contained in the vials. A period of 9–10 min was required for the RFEM to reach 121 °C, as reported by Kasinos et al. [15]. After 30 min in the oil bath, the samples were immediately taken out and cooled in cold water.

#### 2.8.3. Particle Size and Viscosity Measurement

As described previously [16], the volume-weighted average particle size of the RFEM emulsions was measured using a Mastersizer 3000 (Malvern Instruments Ltd., Malvern, UK). The refractive index of the dispersed phase (sunflower oil/protein) was set to 1.47, with an absorption index of 0.01. The continuous phase (water) had a refractive index of 1.330. The stirring speed was set as 1000 rpm. The obscuration for samples was around 10%. No ultrasonication or SDS was used during sample preparation or measurement.

Viscosity measurements of RFEM emulsions after sterilisation were performed using a programmable LV-DV-II+ viscometer (Brookfield, Stoughton, MA, USA). Spindle 34 (immersed directly into the glass vials) was used with a shear rate that ranged from 16 to 52 s^−1^. The consistency coefficient (*K*) was derived by fitting the relationship between the shear stress ( τ) and the shear rate ( γ) to a power law:

(5) $$\tau =K\cdot \gamma^{n}$$

As the apparent viscosity μ is obtained as the ratio of shear stress *τ* (in Pa) to shear rate *γ* (in s^−1^), this power law equation can be rewritten as

(6) $$\mu =K\cdot \gamma^{n-1}$$

where *n* represents the flow behaviour index.

### 2.9. Statistical Analysis

All experiments and incubation of SMP were performed in three independent replicates conducted at different times. A multiple-comparison test (one-way ANOVA) with a post hoc test (Tukey) was applied to analyse the significance of the data after normality check (*n* = 3). Excel 2010 and GraphPad Prism 10 were used to calculate the mean and standard deviation and to draw figures. A value of *p* < 0.05 was considered significant.

## 3. Results and Discussion

### 3.1. Colour Development

**Figure 1:**
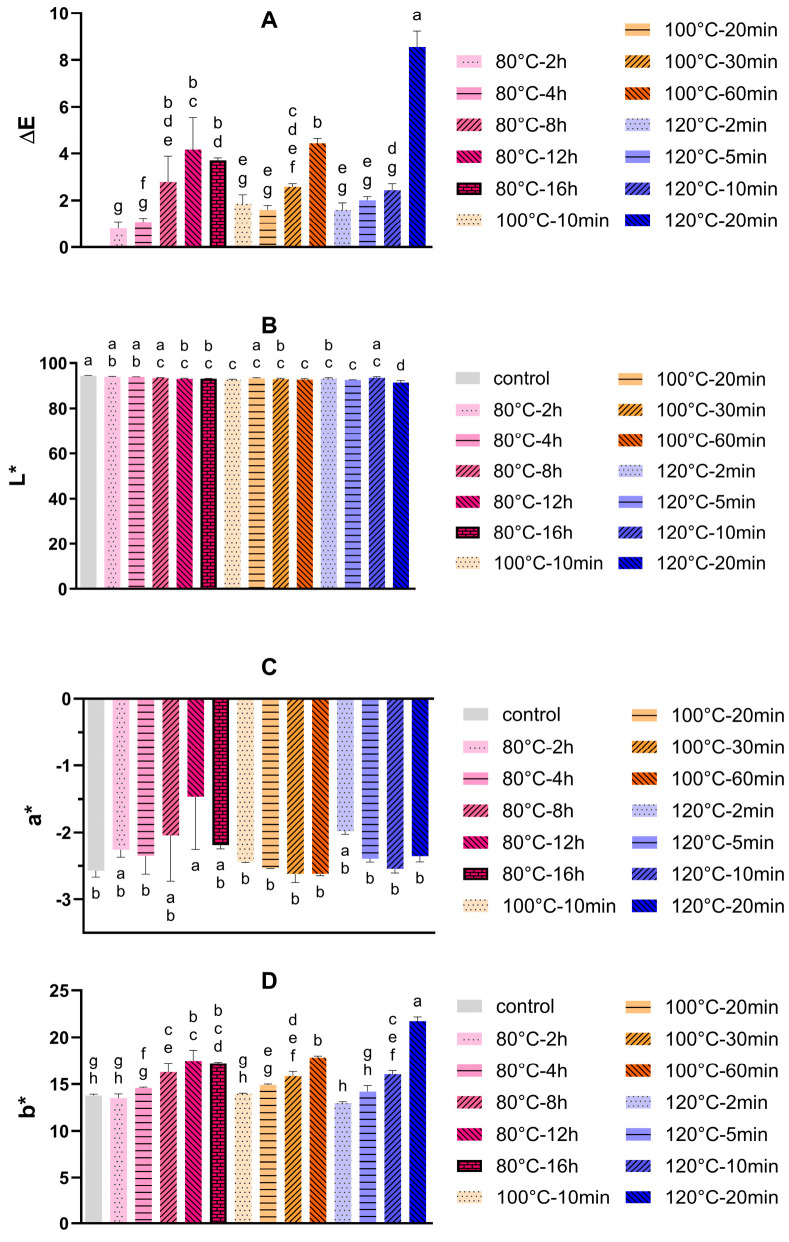
Colour development of SMP as indicated by *ΔE* (**A**), lightness (L*) (**B**), redness (a*) (**C**), and yellowness (b*) (**D**) after dry heating at different time–temperature combinations and a low relative humidity (<10%) (*n* = 3). Values are presented as mean values, and error bars represent the standard deviation of three independent experiments. SMP without dry heat incubation was set as control. Bars with different letters are significantly different (*p* < 0.05, Tukey’s HSD test).

The development of brown colour in SMP can be an indicator of the advanced Maillard reaction, particularly melanoidin formation. As shown in Figure 1A, when incubating at an extremely low relative humidity (<10% RH), only a limited colour change was observed in SMP incubated at 80 °C for 2 h and 4 h, labelled 80 °C-2 h and 80 °C-4 h, respectively. Similarly, the *∆E* values of the 100 °C-10 min, 100 °C-20 min, 120 °C-2 min, and 120 °C-5 min samples were below 2 as well, indicating negligible, i.e., not visible, brown colour development.

Visible browning was observed when the *ΔE* value was higher than 4. For instance, the *∆E* value increased after longer incubation periods at each temperature. The *∆E* values for the 80 °C-12 h, 80 °C-16 h, and 100 °C-60 min samples were around 4, while the *∆E* value of the 120 °C-20 min sample increased to 8, indicating more severe heat damage. In contrast, the lightness value of all SMP samples remained almost unchanged (<2%) after incubation, though there were significant differences between the control and some heat-treated samples, as shown in Figure 1B. For redness (a*), the value of all heat-treated SMP samples tended to increase with a longer incubation time, suggesting that a brown colour tended to appear. However, only the 80 °C-16 h sample presented a significant difference with the control (Figure 1C). The increase in b* value reflected a change in colour towards yellow and brown. As shown in Figure 1D, the 120 °C-20 min sample exhibited the highest increase in b* value (>20) among the tested samples, followed by the 80 °C-12 h, 80 °C-16 h, and 100 °C-60 min samples. The b* results were in line with the *∆E* value, increasing along with the incubation time and temperature.

Sihui Wang et al. also indicated that the browning degree was much lower at low humidity (32%) as compared to 57 or 75% RH after 4 weeks of storage at 37 °C [17]. In this case, no visible colour change was observed in the samples stored at 32% RH, with a *∆E* value around 2, while the *∆E* value for samples incubated at 57 or 75% RH was higher than 10. Although a low humidity can decrease the colour change in SMP, higher temperatures like 120 °C still gave rise to browning. This result can be attributed to the higher reaction rate of the Maillard reaction (MR) at elevated temperatures, even at an extremely low RH.

### 3.2. Development of HMF

During dry heating for SMP, HMF is formed mainly via the 1,2-enolization pathway from the degradation of Amadori products [18]. It can then further react with free amino groups to form melanoidins, which impart a brown colour.

**Figure 2:**
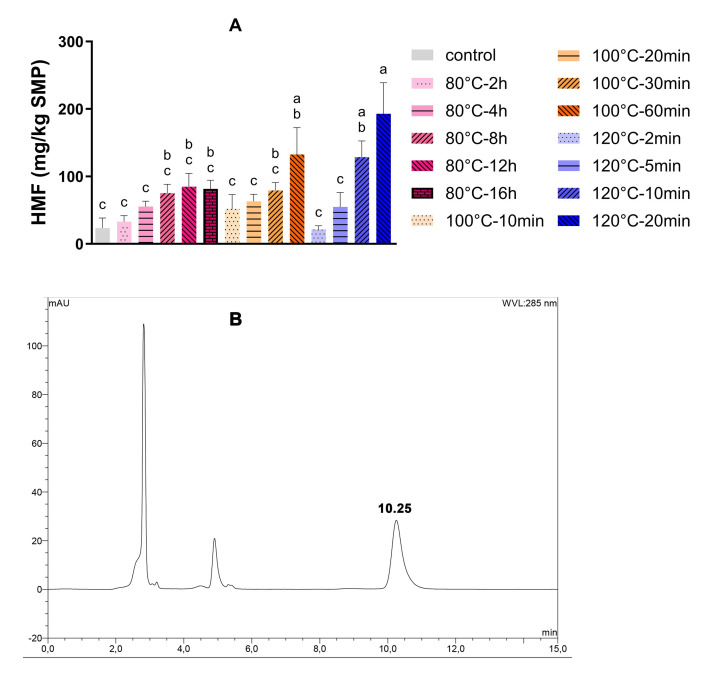
HMF development (**A**) in SMP after dry heating at different time–temperature combinations at a low relative humidity (<10%) (*n* = 3) and chromatogram of HMF (**B**) in an SMP matrix (retention time: 10.25 min). SMP without dry heat incubation was set as the control. Bars with different letters are significantly different (*p* < 0.05, Tukey’s HSD test).

HMF has been generally used as an indicator of the intermediate stage of the Maillard reaction and the severity of heat damage during processing or storage [19]. In this study, the total HMF content was determined in the supernatant after protein precipitation with trichloroacetic acid. As shown in Figure 2A, the HMF concentration of SMP without dry heating (control) was 25 mg HMF/kg SMP. An increase in HMF was observed after dry heating. A higher temperature promoted the formation of HMF, regardless of the extremely low RH.

For instance, the concentration of HMF was 75 mg HMF/kg SMP after 16 h of incubation at 80 °C, whereas only 10 min was enough to reach a concentration of 120 mg HMF/kg SMP when incubated at 120 °C. Different from SMP incubated at 100 or 120 °C, the HMF content reached a plateau around 75 mg HMF/kg SMP after incubation at 80 °C for 8 h, while it kept increasing when incubated at 100 or 120 °C. For instance, the concentration of HMF increased to 200 mg HMF/kg SMP after incubation at 120 °C for 20 min. This might be ascribed to the moderately increased temperature and low water activity in SMP after exposure to such a low-humidity environment for a long time (8 h). The completely dry silica gel absorbed moisture from SMP. Low water activity thus limited the mobility of reactants and decreased the formation of HMF in SMP. In addition, this plateau (from 8 to 16 h) also demonstrates that the formation and degradation of HMF came to a balance. Conversely, the amount of HMF still increased within 60 min and 20 min when incubated at 100 and 120 °C, respectively. At a temperature higher than 100 °C, the high energy input might dominate the formation of HMF regardless of low humidity because the temperatures were higher than the Tg of amorphous lactose in SMP [20], which enabled continuous HMF accumulation without reaching a plateau. It seems that temperature played a major role in the development of HMF, especially high temperatures (≥100 °C).

### 3.3. Protein Solubility of Dry-Heat-Treated SMP Dispersion

Protein solubility is of great importance for commercial SMP, whereby low solubility-induced fouling may impair production equipment The protein solubility of commercial low-heat SMP is normally higher than 90%. Our tested results indicate that the protein solubility of the control SMP was 94%. After dry heat incubation at 80, 100, or 120 °C, all heat-treated SMP samples still maintained high protein solubility (>90%). In our previous study, a dramatic decrease in protein solubility (~60%) in SMP was observed after 16 h of incubation at 74% RH at 60 °C. In the current study, the temperature for dry heating was at least 80 °C, and protein solubility was still quite high (95%) even after incubation for 16 h at 80 °C. These results confirm our hypothesis that a low relative humidity reduces the impairment of protein solubility during dry heating, suggesting limited aggregation. Supplee et al. demonstrated that protein solubility depends on the moisture content in milk powder: a moisture content lower than 3% ensred that the solubility of casein in milk powder remained unchanged for one year. Ideally, proteins retain their original solubility if moisture is entirely excluded [21]. On the contrary, in a humid atmosphere, protein solubility decreases rapidly upon incubation. Le et al. (2011) observed a rapid decrease in solubility when SMP was subjected to 66 or 84% RH at 30 °C for two weeks [10]. Water can be quickly absorbed by hygroscopic powder, which facilitates the unfolding and partial denaturation of whey proteins, especially β-lg. The denatured β-lg tends to release -SH groups, which can interact with each other and with caseins via disulfide bonds, particularly when exposed to higher temperatures. In this regard, protein aggregates comprising denatured whey protein and dissociated casein from micelles lead to the formation of large, insoluble protein complexes.

### 3.4. Sulfhydryl Group Content

**Figure 3:**
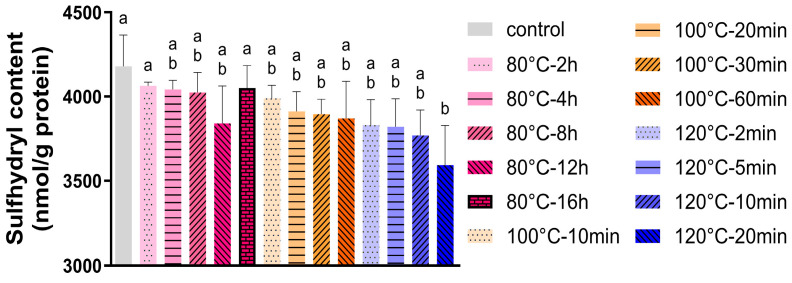
Sulfhydryl content of SMP after dry heating at different time–temperature combinations at a low humidity (<10%) (*n* = 3). SMP without dry heat incubation was set as the control. Bars with different letters are significantly different (*p* < 0.05, Tukey’s HSD test).

In SMP, β-lg is the main source of free sulfhydryl groups (SH) [22]. Heating accelerates the unfolding of β-lg and the exposure of its embedded SH groups, especially when the applied temperature exceeds 80 °C [23]. The exposed SH groups then participate in forming disulphide bonds through oxidation or SH/S-S interchange, whereby the loss of free sulfhydryl groups primarily reflects the former reaction [24,25]. In Figure 3, the sulfhydryl content of SMP without dry heating (control) was around 4000 nmol/g protein. For SMP samples incubated at all tested temperatures, their sulfhydryl content decreased slightly over time. Only the 120 °C-20 min sample exhibited a significant difference with the control. In our previous study [26], however, the sulfhydryl content dropped to 2000 nmol/g protein after dry heating at 80 °C for 2 h at 74% RH, whereby the sulfhydryl content was about half of the value of SMP incubated at 80 °C for 16 h. This suggests that a low moisture content limits protein unfolding even at elevated temperatures (80–120 °C).

Nevertheless, the higher temperature still accelerated the decrease in the sulfhydryl content under low-humidity conditions (<10% RH). The small changes at 120 °C–10–20 min might be due to the longer incubation time at such a high temperature and the original moisture in SMP, which may not have been completely absorbed by the dry silica gel in a short time (within 20 min) and hence may have enabled partial unfolding of β-lg and exposure of embedded sulfhydryl groups.

### 3.5. Protein Carbonyl Group Content

Upon the Maillard reaction and lipid oxidation in milk, reactive dicarbonyl compounds and/or α, β unsaturated aldehydes can be formed, leading to the formation of protein-bound carbonyls [27]. Due to the lack of lipids in SMP, the Maillard reaction is assumed to be the main driver of protein carbonylation during dry heating. Reactive dicarbonyl compounds are mainly formed through fragmentation and dehydration of Amadori products in the intermediate stage of the Maillard reaction [5]. These reactive dicarbonyl compounds can lead to protein crosslinking in the advanced stage via the amino-carbonyl reaction [13] and finally affect the functionality of the products, including their heat stability.

**Figure 4:**
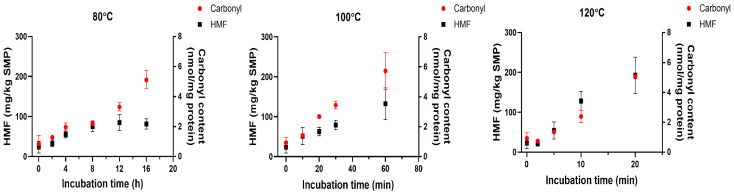
Time-aligned comparison of HMF and protein carbonyl content at each incubation temperature of SMP after dry heating at different time–temperature combinations at a low humidity (<10%) (*n* = 3). SMP without dry heat incubation was set as the control.

In Figure 4, the protein carbonyl content of SMP without dry heating (control) was around 1 nmol/mg protein. Similarly to the above chemical reactions, a higher temperature accelerated the generation of carbonyl compounds. For instance, when incubated at 80 °C, it took 16 h for SMP to produce protein carbonyls with a concentration of 5 nmol/mg protein, while only 60 and 20 min was enough at 100 and 120 °C, respectively. The carbonyl content in the tested SMP samples was below 6 nmol/mg protein at each temperature. This can be attributed to the low relative humidity (RH), which limits the mobility of the reactants as mentioned above. It has been reported by Zhao et al. that SMP developed a high carbonyl content (14 nmol/mg protein) when subjected to a high RH, like 74% RH, at 80 °C for 4 h [26]. The protein carbonyl content upon incubation at 74% RH was seven times higher than the present result at extremely low relative humidity (<10% RH), which highlights the critical influence of relative humidity on the Maillard reaction, whereby low humidity may greatly reduce the formation of protein carbonyls.

Different from the HMF results, the carbonyl content increased linearly over time at all tested temperatures without a plateau. This can be attributed to the fact that reactive carbonyls derived from Amadori degradation can continuously react with free amino groups, and the produced protein carbonyls are chemically stable, while HMF can be degraded to form advanced Maillard reaction products, as mentioned in Section 3.2. Interestingly, the carbonyl content in SMP was comparable at the selected longest incubation periods at all tested temperatures, indicating a similar carbonylation level. On the contrary, more HMF was accumulated at higher temperatures at the selected longest incubation periods (Figure 4). It seems that these two parameters presented different dependencies to temperature because of their different reaction pathways.

### 3.6. Heat Stability of RFEM

**Figure 5:**
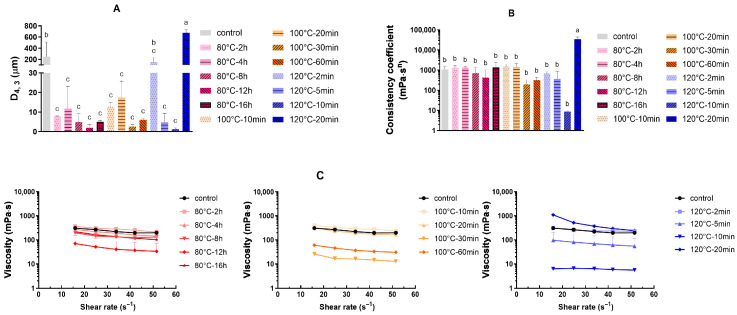
Volume-weighted average droplet diameter (*D_4,3_*) (**A**), consistency coefficient (**B**), and viscosity flow curves (**C**) of the RFEM formulated with dry-heated SMP (with varying dry heat incubation temperatures and durations) after sterilisation at 120 °C for 30 min (*n* = 3). SMP without dry heat incubation was set as the control. Bars with different letters are significantly different (*p* < 0.05, Tukey’s HSD test).

The heat stability of SMP-derived recombined filled evaporated milk emulsions (RFEM) with or without dry heat pretreatment was evaluated via particle size and viscosity measurement of RFEM samples subjected to sterilisation at 120 °C for 30 min. Before heating at 120 °C, all RFEM samples had a homogeneous liquid structure. The volume-weighted average particle diameter before heat treatment was below 1 μm. After heating at 120 °C for 30 min, as shown in Figure 5, the particle size and viscosity of RFEM derived from the original SMP (without dry heating) increased substantially (i.e., *D_4,3_* > 200 μm and consistency coefficient > 1000 mPa·s^n^), and visible coagulation with a gel-like structure was observed.

As compared to the original sample without dry heating, improved heat stability was clearly observed in RFEM derived from SMP upon dry-heat processing at different temperatures, except the SMP samples incubated at 120 °C for 2 min or 20 min. As shown in Figure 5A, most RFEM samples derived from heat-treated SMP presented a 10-fold smaller *D_4,3_* (<20 μm) as compared to the control (200 μm), while the RFEM derived from SMP incubated at 120 °C for 2 or 20 min (i.e., 120 °C-2 min and 120 °C-20 min, respectively) exhibited a much higher *D_4,3_* (200 μm and 600 μm, respectively). Despite subtle overall trends, the post-sterilisation stability of RFEM was influenced by both dry-heating duration and temperature. This is exemplified by the behaviour at 120 °C, where the *D_4,3_* value decreased to 1 µm with an increasing incubation time up to 10 min yet escalated to 600 µm after a 20 min dry-heat incubation period. At lower temperatures (80 and 100 °C), achieving a small *D_4,3_* (<5 μm) required a longer dry-heating duration, and the operational window for improving the heat stability of RFEM was correspondingly broader. The highest RFEM heat stability was observed for RFEM containing SMP upon dry heat pre-treatment for 10 min at 120 °C, leading to a *D_4,3_* value below 1 μm and a consistency coefficient below 10 mPa·s^n^ (Figure 5B). Although the remaining heat-treated SMP derived RFEMs presented a lower *D_4,3_* than the control, even lower than 10 μm, their consistency coefficients were still higher than 100 mPa·s^n^ (Figure 5). For instance, the *D_4,3_* value of the 80 °C-12 h sample was 2 μm, while its consistency coefficient was around 100 mPa·s^n^, which was 10 times larger than the RFEM before the sterilisation. This increased viscosity (Figure 5C) could be attributed to the formation of aggregated protein particles (<2 μm) after sterilisation, which lower the flow behaviour index (*n*), as presented in Table 2. The 120 °C-20 min sample also showed a gel-like structure, while the other samples with a consistency coefficient higher than 100 mPa·s^n^ did not exhibit a gel-like structure, but were flowable, viscous liquids with aggregates. The considerable variance in *D_4,3_* and consistency coefficient values observed across most samples can be attributed to heterogeneity in the extent of glycation among replicates. Nevertheless, the tendency of the consistency coefficient and viscosity flow curves (Figure 5C) as a function of the dry heat incubation time was in line with the D_4,3_ results at all tested temperatures.

For SMP incubated at 120 °C, the *D_4,3_* value and consistency coefficient of RFEM firstly decreased as a function of the dry heat incubation time and then tended to increase after incubation for a specific duration, while at lower temperatures (80 and 100 °C), the increase in *D_4,3_* value and consistency coefficient of RFEM after a longer incubation period in our tested conditions (up to 16 h and 60 min, respectively) was not significant (Figure 5A). A higher incubation temperature induced a narrower incubation window for improved heat stability of RFEM. This phenomenon is consistent with the results of our previous studies [26,28]. Neither a low nor a high degree of Maillard reaction could enhance the heat stability of the derived RFEM, whereas only SMP with a moderate glycation degree displayed high heat stability.

Glycation of milk proteins with lactose upon dry heat incubation of SMP was assumed to be the key process leading to the improved heat stability of the end products (RFEM) [29]. Additionally, the increased surface hydrophilicity imparted by glycation may enhance protein hydration, thereby improving the colloidal stability and suppressing droplet aggregation in emulsions [30]. However, excessive heating could promote the denaturation of the whey proteins, protein cross-linking via disulfide and non-disulfide covalent bonds, and aggregation induced by the advanced Maillard reaction, as well as other unwanted side effects [3]. Hence, it is necessary to modulate glycation and prevent the advanced Maillard reaction stage, even at extremely low relative humidity.

## 4. Conclusions

This study aimed to improve the heat stability of SMP-derived recombined filled evaporated milk emulsions (RFEM) with fewer side effects, such as low solubility, brown colour formation, and formation of other advanced Maillard reaction products. A completely dry environment (<10% RH) was used during dry heating of skim milk powder (SMP). Hereby, higher temperatures (80, 100, and 120 °C) were applied to induce and accelerate the glycation reaction between free amino groups and lactose in SMP. Improved heat stability was observed in SMP upon dry heating under low RH (<10%) across all temperatures at specific incubation periods. Specifically, SMP upon dry heating for only 10 min at 120 °C (120 °C-10 min) acquired the highest thermal stability: its derived RFEM maintained a liquid structure with a *D_4,3_* value below 1 μm and consistency coefficients below 10 mPa·s^n^. In addition, the protein solubility and colour of the 120 °C-10 min sample also remained largely unchanged. Hence, high-temperature dry heating at low humidity is an effective strategy for enhancing the heat stability of RFEM derived from dry-heat-treated SMP. Thus, undesirable effects such as protein carbonyls and browning can be minimised compared to the results of a previous study using dry heating at lower temperatures and higher RH, such as 60 °C and 74% RH. As non-disulfide crosslinks were not evaluated in this study, further investigation into the formation of AGEs in dry-heat-treated SMP will be conducted in our future studies. Overall, this protocol might help the dairy industry to produce heat-stable milk powders.

## Figures and Tables

**Table 1:** Dry heat incubation periods as a function of the incubation temperature.

80 °C	100 °C	120 °C
2 h	10 min	2 min
4 h	20 min	5 min
8 h	30 min	10 min
12 h	60 min	20 min
16 h		

**Table 2:** Consistency coefficient (*K*) and flow behaviour index (*n*) of the RFEM emulsions formulated with dry-heated SMP (with varying dry heat incubation temperatures and durations) after sterilisation at 120 °C for 30 min (*n* = 3).

Incubation Temperature	80 °C			90 °C			100 °C	
Time (h)	*K* (mPa⋅s^n^)	*n*	Time (min)	*K* (mPa⋅s^n^)	*n*	Time (min)	K (mPa⋅s^n^)	n
2	1320 ± 330	0.55 ± 0.10	0	1090 ± 320	0.56 ± 0.12	0	1090 ± 320	0.56 ± 0.12
4	1330 ± 140	0.43 ± 0.04	10	1490 ± 450	0.55 ± 0.15	2	673 ± 140	0.72 ± 0.11
8	710 ± 530	0.49 ± 0.21	20	1440 ± 580	0.46 ± 0.09	5	370 ± 410	0.51 ± 0.03
12	400 ± 500	0.43 ± 0.12	30	199 ± 110	0.43 ± 0.07	10	8.9 ± 0.6	0.90 ± 0.01
16	1350 ± 870	0.34 ± 0.05	60	320 ± 99	0.41 ± 0.09	20	34,800 ± 7900	0.27 ± 0.04

## Data Availability

The original contributions presented in this study are included in the article. Further inquiries can be directed to the corresponding author.

## Abbreviations

The following abbreviations are used in this manuscript:

MR	Maillard reaction
MRPs	Maillard reaction products
AGEs	Advanced glycation end-products
SMP	Skim milk powder
RH	Relative humidity
RFEM	Recombined filled evaporated milk
HMF	Hydroxymethylfurfural
